# Non-Linear Viscoelastic Modeling of PVA Gel Electrolytes for Structural Supercapacitors

**DOI:** 10.3390/ma19173610

**Published:** 2026-08-25

**Authors:** Rafael Schelkow, Davood Peyrow Hedayati, Livia Melina Doß, Robert Böhm

**Affiliations:** Advanced Materials and Structures Lab (AMSL), Faculty of Engineering, Leipzig University of Applied Sciences (HTWK Leipzig), 04277 Leipzig, Germany; davood.peyrow_hedayati@htwk-leipzig.de (D.P.H.); robert.boehm@htwk-leipzig.de (R.B.)

**Keywords:** structural supercapacitor, gel polymer electrolyte, viscoelasticity, constitutive modeling, relaxation test

## Abstract

Knowledge of the time-dependent behavior of polyvinyl alcohol (PVA) gel polymer electrolytes (GPEs) is essential for their application in structural supercapacitors (SSCs) at lower degrees of integration (DoI). Due to the soft, viscoelastic nature of GPEs, their mechanical response under compressive and transient loading is critical for maintaining necessary long-term electrode contact. To enable reliable structural design configurations, a tailored PVA GPE, prepared via a freeze–thaw method, was examined using compressive stress-relaxation testing. The mechanical response was modeled using a novel, non-linear viscoelastic constitutive framework, which couples a time-dependent elastic modulus for the non-linear loading phase with a Prony series for accurate relaxation prediction. The parameters for this practical framework were successfully derived from one single stress-relaxation experiment. Experimental results confirmed pronounced viscoelastic relaxation and up to 10% cyclic hardening. Implemented via finite-element method (FEM) analysis, the non-linear model achieved high accuracy (0.8% average relative deviation), significantly outperforming a linearized model (1.36% deviation). This validated framework is crucial for optimizing the mechanical stability of SSC assemblies and predicting the GPE’s short-time response under transient loading events.

## 1. Introduction

The ongoing global energy transition and the associated shift away from fossil fuels have placed increasing demands on energy storage technologies. Modern electric vehicles, portable electronics, and smart grid applications require power sources that are not only efficient and safe but also lightweight and compact [1,2]. One of the most promising solutions to meet these increasingly stringent requirements is the concept of multifunctional energy storage composites (MESCs) [3,4], which integrates the energy device directly into the mechanical structure of a system.

**Figure 1:**
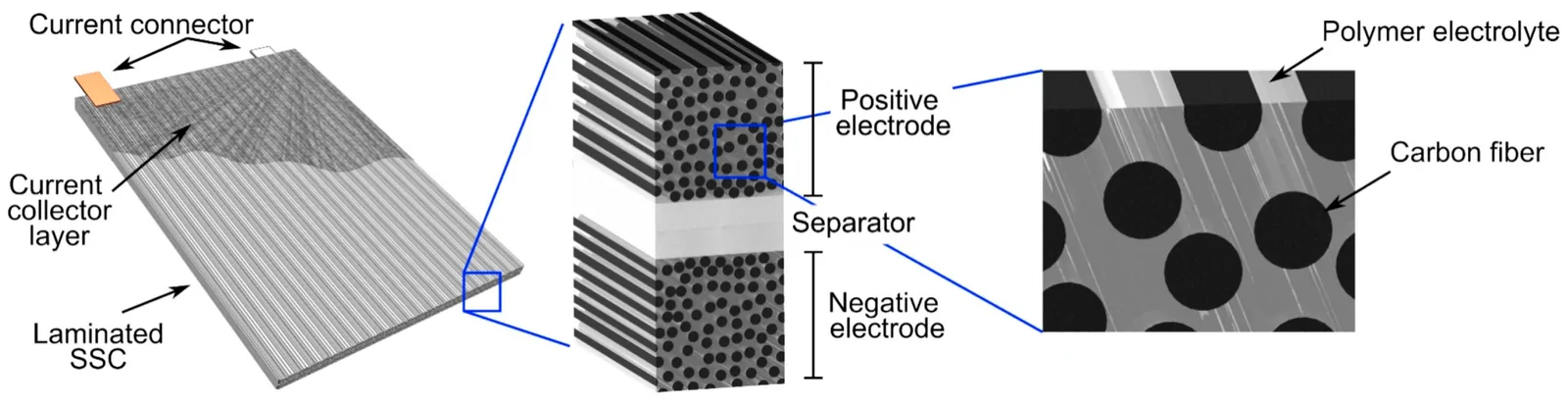
Schematic of a carbon fiber reinforced structural supercapacitor (SSC) structure [7].

Structural supercapacitors (SSCs) represent a key class of MESC devices. These are advanced multifunctional composites, often based on carbon-fiber-reinforced polymers, designed to simultaneously store electrical energy and bear mechanical loads (Figure 1) [5]. This dual functionality offers compelling system-level mass and volume savings by eliminating parasitic weight associated with conventional housings and enclosures.

The primary technical challenge in developing high-performance SSCs lies in the inherent material trade-off. This means components must be optimized for both structural integrity (requiring high stiffness and strength) and electrochemical performance (requiring rapid ion transport, typically achieved with softer, more fluid components) [6,7]. This conflict is particularly pronounced in the design of the electrolyte [8].

To categorize and strategize around this trade-off, the field of MESCs employs the Degree of Integration (DoI) framework [9]. Designs targeting the highest DoI levels (e.g., DoI III and IV) require that every material component, including the electrolyte, possesses high mechanical stiffness, often leading to the selection of solid polymer electrolytes (SPEs) [10]. However, while structurally robust, these high-DoI SPEs typically suffer from lower ionic conductivity, which restricts their power density and overall electrochemical performance.

Our work strategically addresses this limitation by focusing on SSC architectures compatible with Low to Intermediate DoI (e.g., DoI I or II). In these systems, the primary mechanical load is borne by high-performance external reinforcement (such as specialized fiber-reinforced laminates or robust housing) and protective sealer layers. This design decouples the functions, creating an opportunity to use high-performance, inherently softer electrochemical materials [7].

In this context, we employ a high-performance polyvinyl alcohol (PVA) gel polymer electrolyte (GPE). PVA GPEs, prepared via a freeze–thaw fabrication step, offer superior ionic conductivity and electrochemical stability compared to most rigid structural SPEs [11]. By utilizing the GPE at these lower integration levels, where it is confined and protected by a stiff matrix, we leverage its excellent electrochemical properties without relying on it to bear the primary structural load.

However, even in low-DoI assemblies, the electrolyte is subjected to significant internal compressive stresses during manufacturing and transient loading events throughout the device’s service life [12]. In solid-state energy-storage systems, maintaining intimate and stable contact between the electrolyte and the electrodes is particularly critical, because ionic transport occurs primarily across the electrode–electrolyte interface. Any loss of contact or interfacial delamination can increase the interfacial resistance, reduce ion-accessible surface area, and consequently degrade power capability, energy efficiency, and cycle life [5]. PVA-based gel electrolytes exhibit a soft, highly nonlinear, and viscoelastic mechanical response characterized by time-dependent deformation and stress relaxation under sustained loading. Therefore, accurately modeling this behavior is essential for predicting the evolution of contact pressure at the electrode–electrolyte interface, preserving interfacial integrity, and assessing long-term mechanical degradation and fatigue [13].

Numerous studies have investigated the time-dependent mechanical response of PVA hydrogels, particularly in the context of tissue engineering, where they are often combined with various reinforcement architectures [14,15,16]. Furthermore, a considerable number of experimental and analytical studies on hydrogels focus on their viscoelastic behavior [17,18,19,20]. For hydrogels designed for use as electrolytes, studies have analyzed cellulose-based [21] as well as other formulations [22,23] to investigate the interdependence of mechanical and electrochemical properties. However, comprehensive investigations explicitly targeting the non-linear viscoelastic behavior of hydrogel electrolytes, especially those tailored for structural supercapacitors, remain scarce. Existing models often employ simplified linear viscoelastic assumptions [24] or require extensive experimental campaigns and complex parameter identification procedures [25,26]. Moreover, cyclic hardening effects observed under repeated loading, critical for long-term structural prediction, have received limited attention.

Despite the critical role of GPE mechanics in the long-term reliability of structural energy-storage devices, the mechanical behavior of these materials remains comparatively underexplored in the energy-storage literature. Most studies primarily report elastic properties derived from monotonic tensile or compression tests, or evaluate mechanical performance at a single loading rate, while the constitutive models employed often neglect the coupled nonlinear and time-dependent viscoelastic response that is intrinsic to hydrated polymer networks [13,27,28]. Consequently, stress relaxation, creep, and the evolution of electrode–electrolyte contact pressure under sustained or cyclic loading are rarely incorporated into predictive numerical models, limiting their ability to accurately represent the mechanical behavior of GPEs during service. This paper closes that gap by presenting a comprehensive non-linear, short-time viscoelastic constitutive model for a tailored PVA hydrogel electrolyte.

In this context, the objective of this work is to establish a constitutive model capable of describing the nonlinear and time-dependent mechanical response of a PVA-based gel polymer electrolyte under compressive loading. To this end, controlled stress-relaxation experiments are performed to characterize the material behavior, and the resulting data are used to calibrate a nonlinear viscoelastic model that combines a nonlinear elastic response during loading with a Prony-series representation of stress relaxation. The calibrated model is subsequently implemented in a finite-element analysis (FEA) framework to enable predictive simulations of gel polymer electrolytes and to facilitate the mechanical design and optimization of low-degree-of-integration structural supercapacitor assemblies.

## 2. Materials and Methods

### 2.1. Hydrogel Preparation

**Figure 2:**
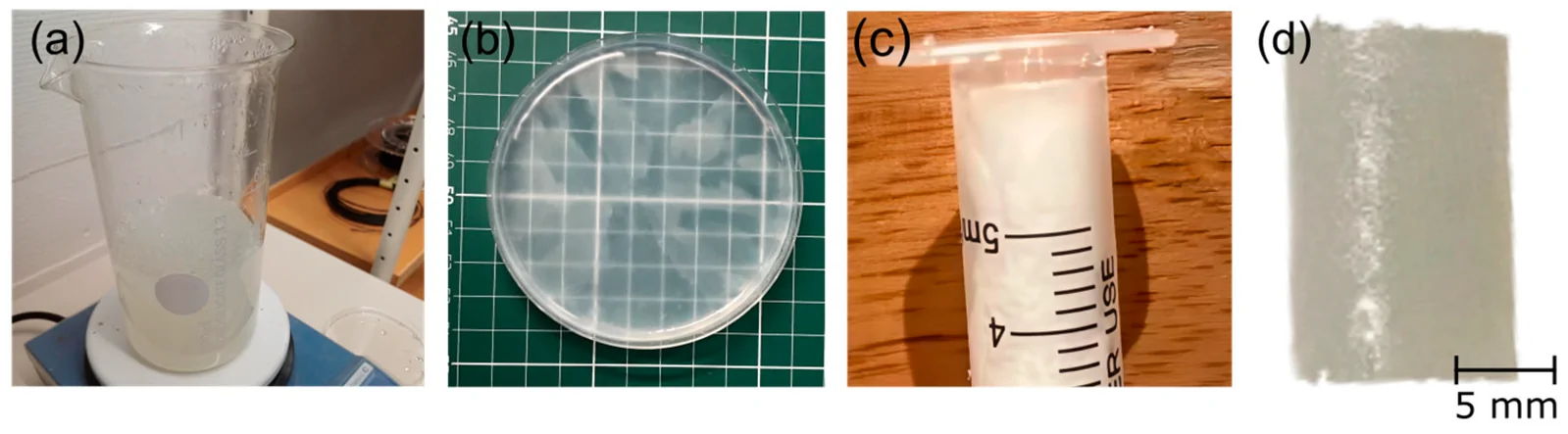
(**a**) Solved PVA in water; (**b**) PVA-water solvent in a Petri dish after one freeze cycle; (**c**) PVA-gel in a syringe for curing; (**d**) finished PVA-gel sample.

The preparation of the GPE was carried out following the procedure described by Nafo et al. [24]. The GPE consisted of 20 wt.% PVA and 80 wt.% water to maximize electrochemical performance. Accordingly, a total mixture of 20 g of PVA and 80 g of water was prepared. We also used 1 M NaCl salt. The PVA powder (Thermo Fisher Scientific Chemicals, Waltham, MA, USA) with a high molecular weight grade with 98–99% hydrolysis was dissolved in hot water at 80 °C under continuous stirring (Figure 2a). The samples were then frozen for 3.5 h in a deep freezer (Liebherr-Hausgeräte GmbH, Ochsenhausen, Germany) (Figure 2b). After this period, they were thawed at room temperature for 2 h and frozen again for another 3.5 h. The freeze–thaw cross-linking step was repeated three times. To produce five cylindrical samples, syringe barrels sealed at the tip were used. The prepared solution was filled into the syringes and subsequently sealed with the plunger (Figure 2c). After completion of the cross-linking steps, the samples were unmolded and cut to size to ensure planar contact surfaces (Figure 2d). The resulting samples exhibited a rubber-like nature with a rough surface.

The samples’ dimensions are given in Table 1. While samples’ dimensions had a relatively high tolerance due to the nature of the hydrogel, the aspect ratio remained within the acceptable range.

### 2.2. Stress Relaxation Test

**Figure 3:**
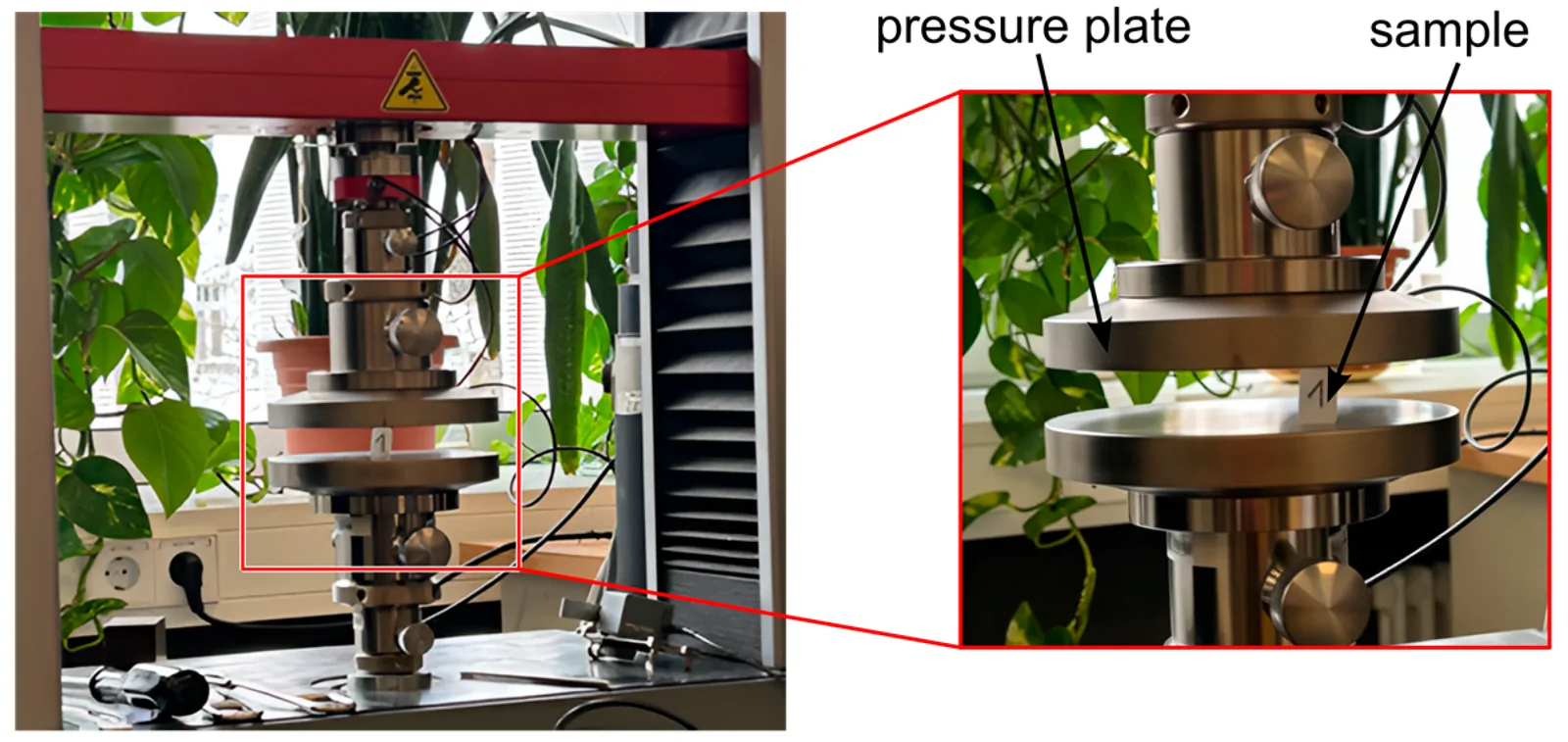
Testing setup for the relaxation test.

The stress relaxation tests were performed under deformation-controlled conditions (Table 1). The testing was conducted using a universal testing machine (ZwickRoell GmbH & Co. KG, Ulm, Germany), which provided the necessary precision for the applied load and displacement control. The setup, including the sample fixture and machine components, is shown in Figure 3.

**Figure 4:**
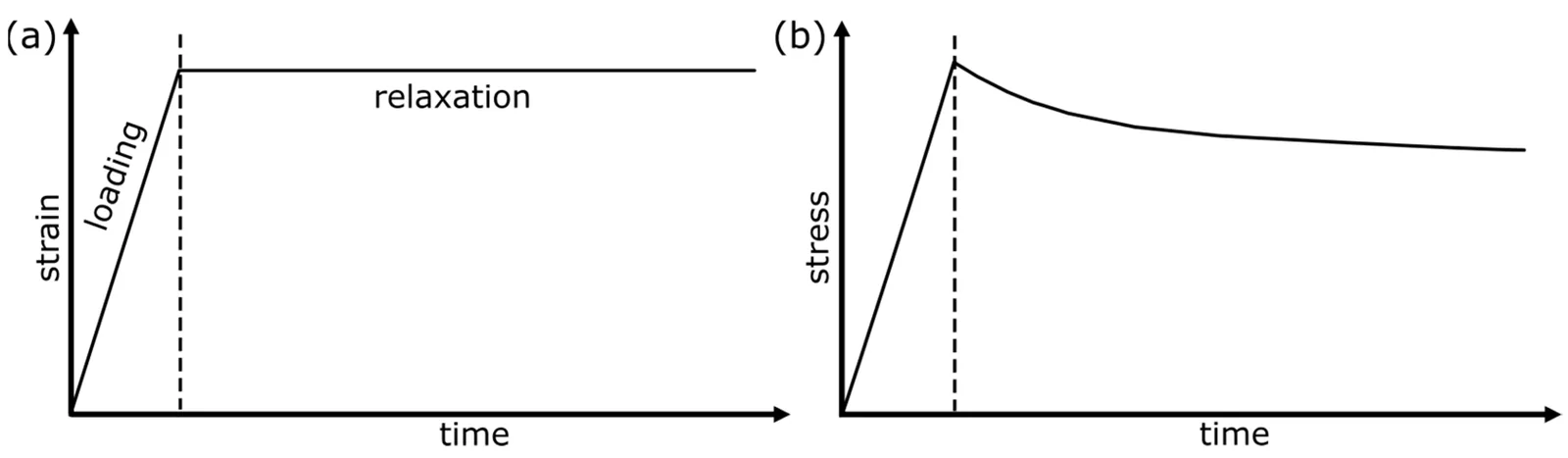
Schematic of one test cycle: (**a**) applied strain over time and (**b**) expected stress response over time.

The relaxation procedure involved subjecting each cylindrical sample to a defined loading cycle (C), which was repeated three times (C1 to C3) to ensure the reproducibility of the measurements and to account for any potential initial setup or material deviations. Engineering stress and engineering strain were used throughout the experimental evaluation. The engineering stress was calculated by dividing the measured compressive force by the initial cross-sectional area of the specimen [29], while the engineering strain was determined as the ratio of the applied displacement to the initial specimen height [29]. Changes in the specimen cross-sectional area during compression were not considered in the stress calculation. A schematic representation of one complete test cycle, showing the applied strain profile over time and the expected resulting stress response without the unloading phase, is presented in Figure 4.

Before each compression cycle, a preload force of 0.5 N was applied to establish stable and reproducible contact between the compression platen and the specimen. The preload was selected to minimize uncertainties associated with initial contact detection and surface irregularities while remaining sufficiently small to avoid significant pre-deformation or influencing the subsequent stress-relaxation response. The specimen was then compressed to the prescribed displacement and unloaded at a rate of 30 mm/min. A recovery period of 5 s was applied between consecutive cycles, after which the 0.5 N preload was reapplied. The tests were conducted at an ambient temperature of 20 °C, and force and displacement data were recorded at a sampling frequency of 500 Hz. The detailed test conditions, including the distinct steps of the loading cycle, are summarized in Table 2.

### 2.3. Constitutive Model and Governing Equations

To model the viscoelastic stress–strain response of the GPE, the following assumptions and simplifications were made: (i) inertial effects and mass-related influences are neglected, and the mechanical response is governed by the quasi-static equilibrium equation; (ii) the material behavior is assumed to be isotropic; (iii) the hardening of the material after the first cycle is not captured by the model; (iv) the material exhibits large deformations and is modeled using a coupled constitutive approach, i.e., a time-dependent elastic component captures the non-linear loading phase, and a linear viscoelastic component, described by a Prony series, captures the time-dependent relaxation [30].

Since a quasi-static analysis is performed here and all mass-related influences are not taken into account, the equilibrium reaction is as follows [31]:

(1) ∇·σ=0

The structural deformations are described by the infinitesimal strain tensor ε, which is related to the displacement field *u* by [32,33]:

(2) $$\varepsilon =\frac{1}{2}\left(\nabla u+{\left(\nabla u\right)}^{T}\right)$$

where *u* is the displacement vector. The linear elastic constitutive relationship between the infinitesimal strain tensor ε and the Cauchy stress tensor ***σ*** according to Hooke’s law for isotropic materials is given by [33]

(3) $$\sigma =\lambda_{m}tr\left(\epsilon \right)I+2\mu_{m}\epsilon$$

where the Lamé constants $\lambda_{m}$ and $\mu_{m}$ are derived from the relationship with the modulus of elasticity E and Poisson’s ratio ν as follows [31]:

(4) $$\lambda_{m}=\frac{E\nu }{\left(1+\nu \right)\left(1-2\nu \right)},\;\mu_{m}=\frac{E}{2\left(1+\nu \right)}$$

Substituting into (3) results in

(5) $$\sigma =\frac{E\nu }{\left(1+\nu \right)\left(1-2\nu \right)}tr\left(\epsilon \right)I+\frac{E}{\left(1+\nu \right)}\epsilon$$

In order to incorporate the non-linear behavior in the loading phase, a time-dependent modulus of elasticity E(t) was introduced, which changes the equation as follows:

(6) $$\sigma (t)=\frac{E(t)\nu }{\left(1+\nu \right)\left(1-2\nu \right)}tr\left(\epsilon \right)I+\frac{E(t)}{\left(1+\nu \right)}\epsilon$$

The time-dependent behavior of the material, which describes stress relaxation τ(t), is given as follows for a viscoelastic model [30]:

(7) $$\tau_{t}\left(t\right)=\int_{0}^{t}\mu \left(t-t^{\prime }\right)\dot{\gamma }(t^{\prime }){dt}^{\prime }$$

**Figure 5:**
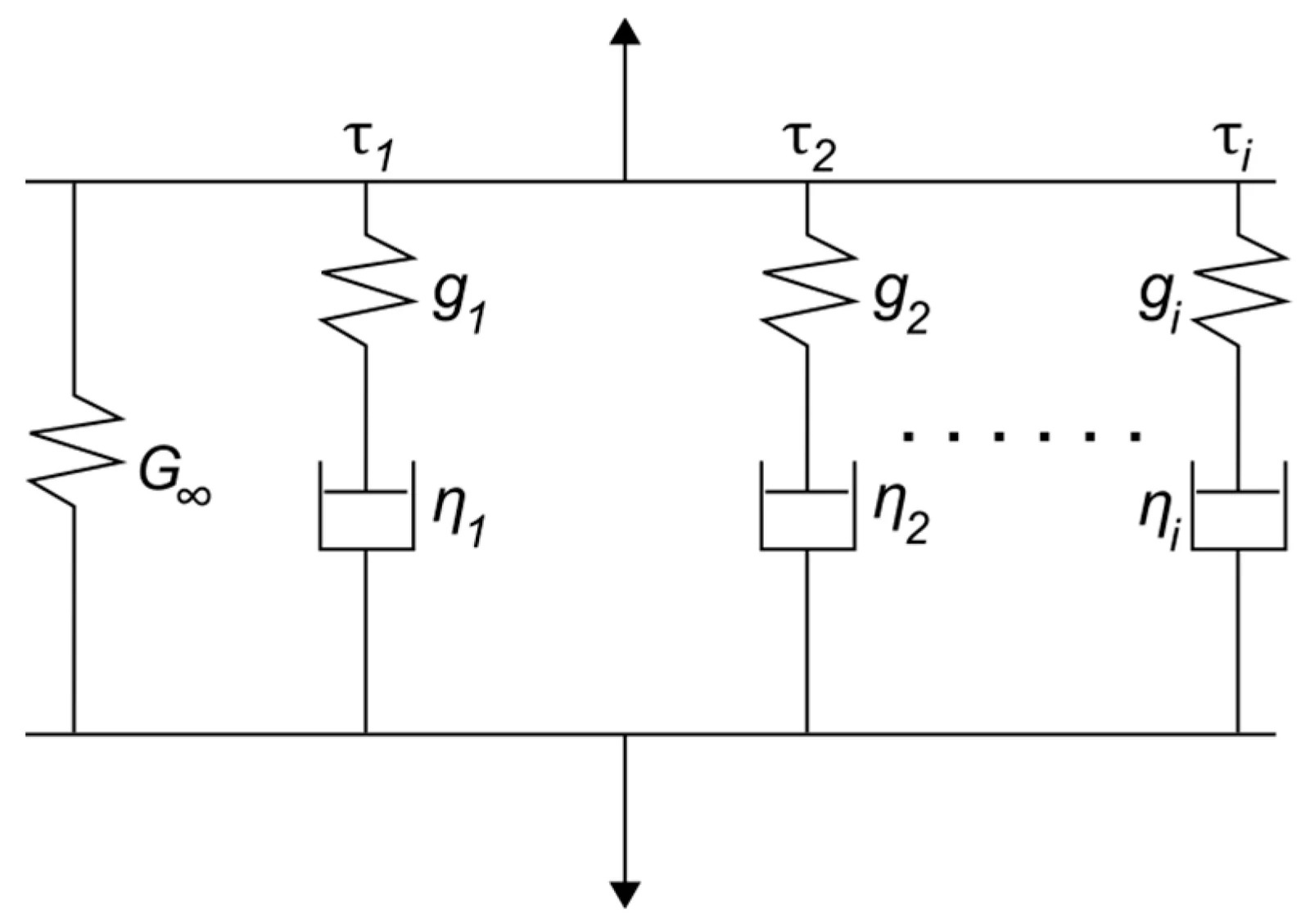
Schematic of the generalized Maxwell model.

Here, $\tau_{t}$ denotes the time-dependent viscous component of the stress, which represents the material’s stress history and should not be confused with the discrete relaxation times $\tau_{i}$ used in the Prony series. In (7), $\dot{\gamma }$ is the strain rate tensor and μ(t) is the time-dependent relaxation modulus. The viscoelastic behavior in the relaxation phase was described using a Prony series. The model allows both the creep process and the stress relaxation to be represented by a sum of exponentially decaying functions. The Prony series can be formulated using the expression [30]

(8) $$\mu \left(t\right)=G_{\infty }+\sum_{i=1}^{N}g_{i}e^{-t/\tau_{i}}$$

with $G_{\text{∞}}$ as the equilibrium modulus and $g_{i}$, $\tau_{i}$ and N as the relative modulus, relaxation time, and relaxation mode, respectively. $\tau_{i}$ is the quotient of the corresponding viscosity $\eta_{i}$ and the modulus $g_{i}$. For this model, it is assumed that the behavior of the material can be modeled by several Maxwell models connected in parallel (see Figure 5) [30]. Each individual element has a relaxation time to which a specific relative modulus belongs. When the terms are added together, the result is the overall viscoelastic behavior.

The parameters were determined by fitting the experimental data from the stress relaxation test using ANSYS (Version 2024 R2, Ansys Inc., Canonsburg, PA, USA). The viscoelastic behavior was implemented using the built-in isotropic linear viscoelastic material model based on a generalized Maxwell formulation. A four-term Prony-series representation was selected as a lowest pragmatic compromise between fitting accuracy and model complexity. The number of terms was determined through an iterative fitting approach. The selected model provided a satisfactory representation of the experimentally observed stress-relaxation response while limiting the number of fitting parameters. The four-term Prony series was defined for the shear relaxation modulus, while the bulk modulus was assumed to remain constant. The instantaneous Young’s modulus was $E_{0}$ = 0.0877 MPa, the corresponding equilibrium Young’s modulus was $E_{\infty }$ = 0.0414 MPa, and a constant Poisson’s ratio of ν=0.35 [34,35] was assumed throughout the simulations. The Prony coefficients were normalized with respect to the instantaneous shear modulus. The following relative shear moduli and relaxation times in Table 3 were used for the calculation with ANSYS. The longest relaxation time and the corresponding shear modulus exceed the holding time of the experiment and should therefore be regarded as an approximation of the long-term relaxation trend rather than as a fully identified relaxation process.

In order to represent the highly non-linear behavior of the loading phase, a phenomenological time-dependent modulus of elasticity E(t) was selected to represent the non-linear behavior of the GPE in the loading phase. The function E(t) was first determined for each loading cycle of the respective sample and then averaged for the individual samples as total moduli. Consequently, E(t) should be interpreted as a constitutive representation valid for the loading conditions considered in this study rather than as a universal intrinsic material property of the PVA hydrogel. The proposed model is therefore applicable to the investigated quasi-static loading conditions at ambient temperature, while its extension to different strain rates or temperatures would require additional experimental characterization. This resulted in the moduli of elasticity for the individual samples in Table 4. For the relative mean deviation (RMD), the following equation was used:

(9) $$\mathrm{RMD}=\frac{1}{n}\sum_{i=1}^{n}\left|\frac{x_{i}-\bar{x}}{\bar{x}}\right|$$

**Figure 6:**
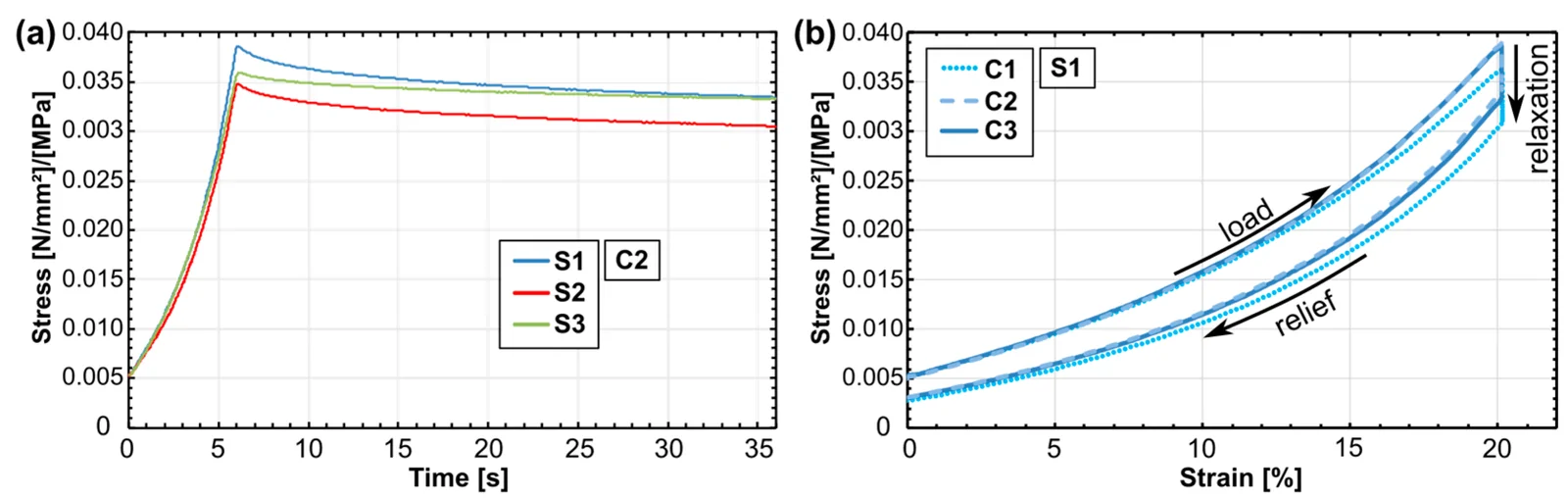
(**a**) Experimental results of all samples during the second cycle; (**b**) stress response of sample 1 (S1) to the applied strain, including the unloading phase across all cycles. S and C refer to sample and cycle, respectively.

A high deviation of the time-dependent E-modulus up to 10% between the values can be seen. This behavior is also reflected in the experimental results. The samples exhibited not only differences between the individual relaxation test cycles, with increased stiffness observed after the first cycle, but also noticeable variability between the samples themselves. A comparison of the samples with each other and their behavior during the cycles can be seen in Figure 6.

### 2.4. FEM Analysis and Boundary Conditions

**Figure 7:**
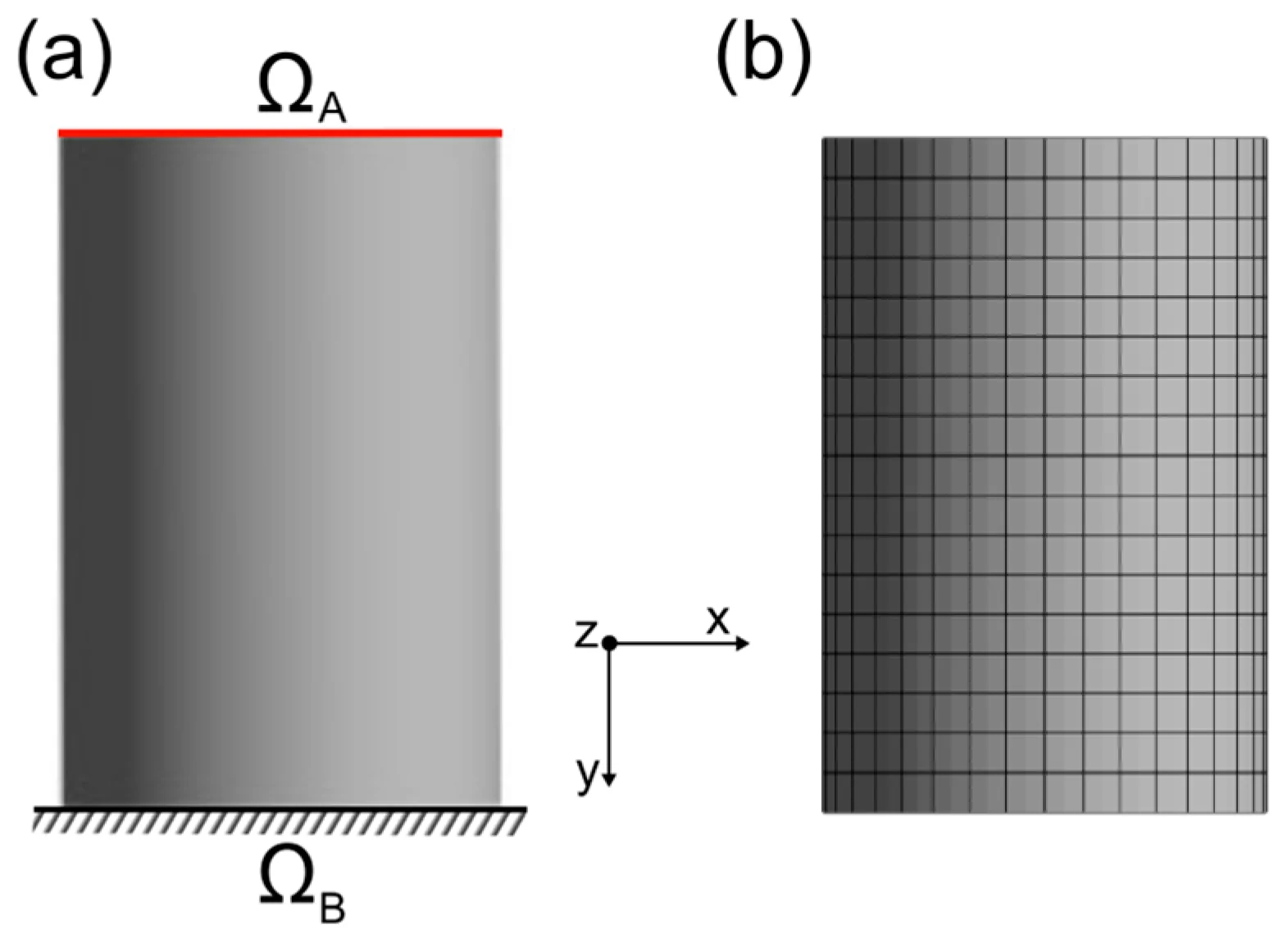
(**a**) Schematic of the simulation geometry and the defined boundary areas; (**b**) mesh used for the computation.

The time-dependent elastic modulus (Table 4) and the identified Prony parameters (Table 3) were implemented in the ANSYS viscoelastic material model. To replicate the experimental procedure described in Section 2.2, the FE simulations were performed by first applying a preload of $F_{\mathrm{pre}}=0.5\;N$; then, the deformation of $u_{\max}=3\;mm$ was applied over a beginning period of $t_{b}=6\;s$, followed by a holding duration of $t_{h}=30\;s$ to replicate the experimental setup (see Section 2.2). For this purpose, all samples were analyzed with respect to their specific initial geometry (Figure 7a). Owing to the cylindrical specimen geometry, a three-dimensional solid finite-element model was developed in ANSYS Mechanical (Version 2024 R2, Ansys Inc., Canonsburg, PA, USA). The mesh was generated using quadratic 20-node SOLID186 solid elements, which allow an accurate representation of the cylindrical geometry through hexahedral and wedge-shaped element configurations where required. An average element size of 1 mm resulted in a total of 3936 elements (Figure 7b). A quantitative mesh convergence analysis was performed by comparing the reaction force response obtained with different mesh densities. Mesh sizes of 2.0 mm, 1.5 mm, 1.0 mm, and 0.5 mm were evaluated. The maximum reaction force differed by less than 0.6% between the coarsest and finest mesh, while the selected 1 mm mesh deviated by only 0.21% compared with the 0.5 mm mesh. Therefore, the selected mesh density was considered sufficient to obtain mesh-independent results. Since the specimen was modeled as a single continuous solid body, no contact interfaces or frictional contact conditions were defined. The lower surface was fixed, while a prescribed displacement was applied to the upper surface. To reproduce the experimental compression setup, lateral displacements in the x- and z-directions were constrained at the specimen surfaces. The large-deformation option was activated to account for geometric nonlinearities during compression, and the analysis was performed using the program-controlled solver settings in ANSYS Mechanical.

As boundary conditions, during loading by means of the pre-force, a combination of Neumann and Dirichlet boundary conditions were set on the boundary surface $\Omega_{A}$ as follows:

(10) $${u_{x,z}=0,\;F_{y}=F_{pre}\;on\;\Omega }_{A}$$

Subsequently, the Neumann boundary condition on the domain $\Omega_{A}$ is replaced by a displacement in the y-direction during the loading and relaxation analysis.

(11) $$u_{x,z}=0,\;u_{y}=\{\begin{array}{cc} u_{max}\frac{t}{t_{b}} & 0\leq t\leq t_{b}\\ u_{max} & t_{b}\leq t\leq t_{b}+t_{h} \end{array}\;on\;\Omega_{A}$$

On the boundary surface $\Omega_{B}$, the Dirichlet boundary condition is formulated as follows:

(12) $${u_{x,y,z}=0\;on\;\Omega }_{B}\;t\in \left[0,t_{b}+t_{h}\right]$$

## 3. Results and Discussion

**Figure 8:**
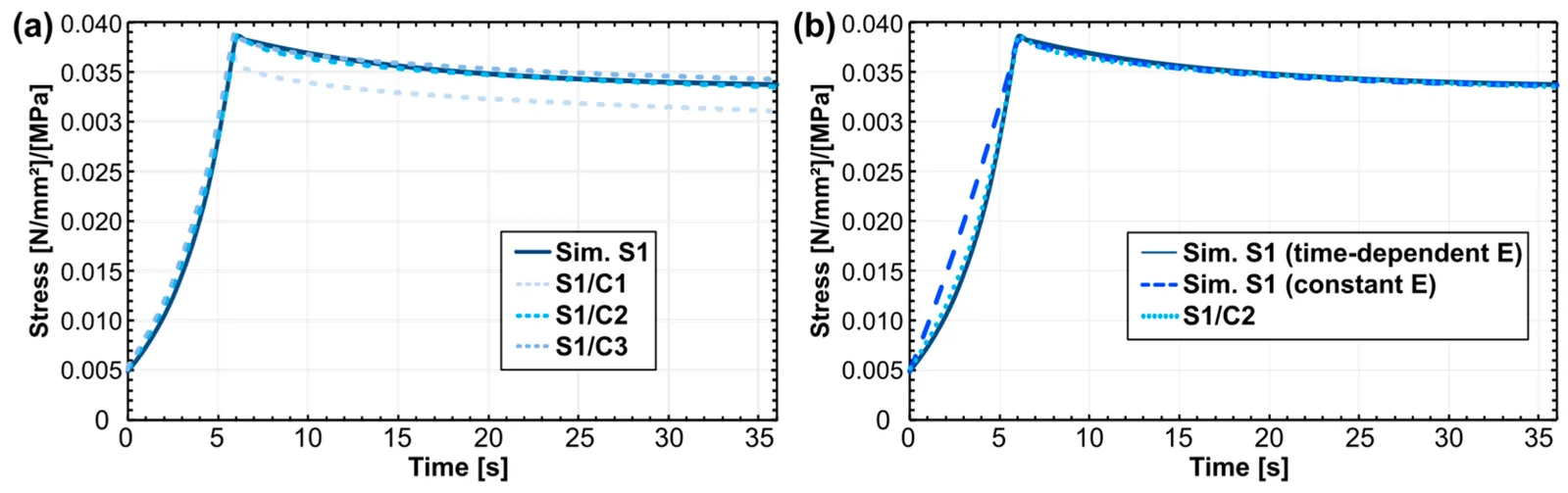
(**a**) Comparative representation of the simulated results for sample 1 and the experimental data over all cycles; (**b**) comparison between the time-dependent approach and the assumption of a constant elastic modulus with respect to the experimental results.

Figure 6 and Figure 8 illustrate the viscoelastic response of the tested samples under cyclic loading and highlight the importance of incorporating time-dependent material properties into the simulation model.

In Figure 6a, the stress–time curves reveal pronounced viscous behavior during the relaxation phase across all three samples, with variations in peak stress and relaxation attributed to material heterogeneity and geometric differences. In Figure 6b, hardening effects become evident after the first cycle, while some energy dissipation could be observed due to possible internal friction upon unloading, which reflects the combined viscous-elastic nature of the material.

The comparison in Figure 8a demonstrates that the simulation provides a good fit to the experimental data after the first cycle. Quantitatively, the average relative deviation between the model and the experimental data for Sample 1 after one cycle is 0.8% over the entire test cycle and 3.3% during the loading phase, confirming the accuracy of the viscoelastic approach. In contrast, Figure 8b shows that the linearized model with a constant Young’s modulus of $E_{const}$ = 0.1504 MPa produces higher deviations of 1.36% and 7% in the loading phase. Direct comparison reveals that the deviations of the linearized model are almost twice those of the variable E-modulus model. Consequently, the introduction of a time-dependent E-modulus substantially improves the predictive capability of the simulation, enabling a more accurate representation of the experimental behavior, particularly during the loading phase.

**Figure 9:**
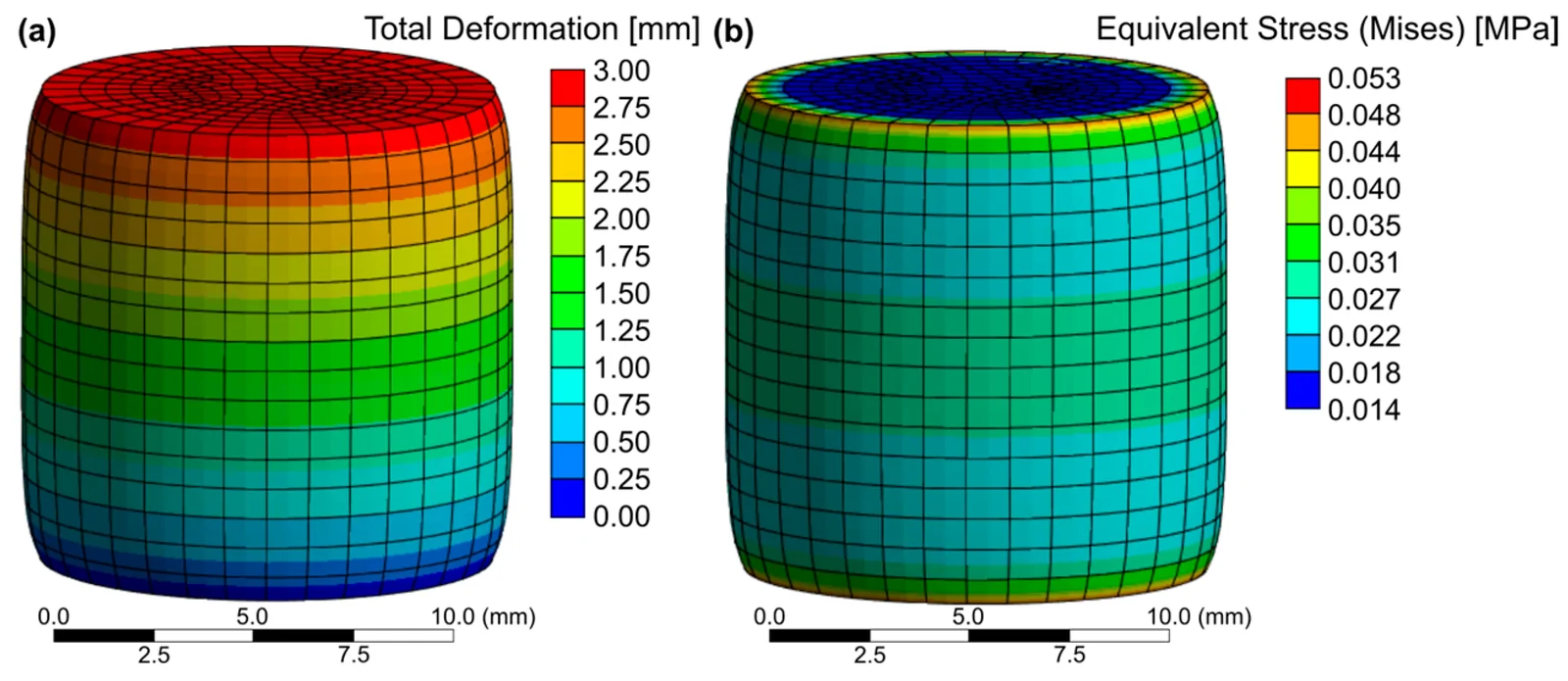
FE simulation results of the GPE (S1) under compression at t=6 s: (**a**) the total deformation and (**b**) von Mises equivalent stress.

Figure 9 visually presents the simulated state of Sample 1 at the end of the loading phase (t = 6 s), right before the stress relaxation phase begins, obtained from the FEM analysis using the coupled non-linear viscoelastic model. By combining a time-dependent modulus with a Prony series or Maxwell models, both the strongly nonlinear loading phase and the subsequent relaxation phase can be accurately represented. This approach enables optimized mechanical design of structural energy storage systems with solid gel electrolytes, particularly with regard to transient loads under constant forces or displacements over the entire service life of the product.

There remains potential for optimization in sample preparation and experimental setup. The manufactured samples exhibited coarse structural elements and fluctuations in dimensional accuracy, leading to measurable deviations in mechanical behavior and indicating that further homogenization of the production process is necessary. Recording the sample cross-section during testing and adjusting the sample geometry toward flatter bodies could reduce unwanted effects such as buckling under compressive loads and thereby improve the reproducibility of the experimental results. The observed hardening effect of up to 10% after the first cycle Figure 6b and the good agreement between simulation and experiment thereafter are important findings, suggesting that the identified material parameters are representative of the hardened/stabilized material state. The four-term Prony series provided a satisfactory representation of the measured relaxation behavior; however, its parameters were identified using an iterative fitting approach rather than a systematic model-selection methodology. In addition, the longest relaxation time slightly exceeded the experimental holding period, indicating that extended stress-relaxation experiments would be beneficial for a more reliable identification of the relaxation behavior and Prony-series parameters.

The 30 s holding period was selected based on preliminary tests and the specific characteristics of the PVA/NaCl gel electrolyte. Longer holding times may introduce additional effects, such as water loss or water diffusion, which could influence the measured mechanical response and potentially affect the electrochemical properties of the electrolyte. The present study therefore focused on the short-term response under conditions intended to avoid such effects. Nevertheless, longer-term stress-relaxation behavior requires further investigation using extended holding periods.

The proposed time-dependent Young’s modulus was likewise identified from a single loading history and should therefore be considered a phenomenological representation applicable to the investigated quasi-static loading conditions at ambient temperature rather than a universal material property. Future investigations at different loading rates and temperatures, as well as studies of the electrochemical properties of hydrogel electrolytes and their correlation with mechanical relaxation behavior, are important for developing a more comprehensive understanding of GPE behavior. Likewise, studying the influence of added salts on mechanical behavior could provide further insight into the material characteristics and support more reliable predictions of SSC performance under sustained loading.

## 4. Conclusions

In conclusion, the developed methodology enables the characterization of the nonlinear and viscoelastic behavior of the investigated PVA/NaCl gel under short-term quasi-static compression using a single stress-relaxation test. The phenomenological constitutive model combines a time-dependent Young’s modulus for the loading response with a Prony series for the relaxation behavior and was successfully implemented in an ANSYS FEM model. The approach provides a practical basis for representing the mechanical response of the investigated GPE under the tested conditions. However, it should not be interpreted as a predictor of long-term material behavior or service life. Future work should focus on extended stress-relaxation experiments, systematic parameter identification, investigations at different loading rates and temperatures, and improved sample preparation.

## Figures and Tables

**Table 1:** Gel polymer electrolyte sample dimensions.

Sample ID [-]	Length *l* [mm]	Diameter *d* [mm]	Aspect Ratio *l*/*d* [-]
S1	14.89	11.37	1.31
S2	14.34	11.20	1.28
S3	16.54	11.30	1.46

**Table 2:** Testing parameters of the relaxation test.

Parameter	Value	Unit
Pre-load Force	0.5	N
Max. Deformation Displacement	3.0	mm
Deformation Un-/Loading Rate	30	mm/min
Stress Relaxation Holding Time	30	s
Recovery Time	5	s
Ambient Temperature	20	°C

**Table 3:** Prony parameters of the GPE for the computation with ANSYS.

Index i [-]	Relative Shear Modulus $g_{i}$ [-]	Relaxation Time $\tau_{i}$ [s]
1	0.463	6.23
2	0.0532	9.16
3	0.0102	19.93
4	0.00147	35.84

**Table 4:** Time-dependent elastic modulus of each sample and the relative mean deviation.

Time, t [s]	$E_{1}(t)$ [MPa]	$E_{2}(t)$ [MPa]	$E_{3}(t)$ [MPa]	RMD [%]
0.5	0.065	0.057	0.076	9.84
0.9	0.066	0.057	0.078	10.43
1.2	0.067	0.059	0.078	9.88
1.6	0.070	0.061	0.081	9.97
2.1	0.073	0.064	0.084	9.58
2.5	0.076	0.067	0.088	9.39
2.8	0.080	0.070	0.092	9.23
3.2	0.084	0.074	0.096	8.78
3.5	0.087	0.078	0.100	8.45
3.8	0.091	0.081	0.103	8.07
4.1	0.095	0.085	0.106	7.80
4.6	0.101	0.090	0.112	7.16
5.0	0.109	0.096	0.118	7.17
5.5	0.118	0.104	0.125	6.91
6.0	0.127	0.112	0.133	6.40

## Data Availability

The original data presented in the study are openly available in Stress Relaxation Test Dataset of Cylindrical PVA Gel Polymer Electrolyte (GPE) Samples at 10.5281/zenodo.21333840.

## Nomenclature

**Symbol**	**Meaning**	**Symbol**	**Meaning**
σ	Stress tensor	μ(t)	Time-dependent relaxation modulus
ϵ	Strain tensor	$G_{\text{∞}}$	Equilibrium modulus
u	Displacement vector	$g_{i}$	Relative modulus
I	Identity matrix	$\tau_{i}$	Relaxation time
$\lambda_{m}$	First Lamé constant	N	Relaxation modes
$\mu_{m}$	Second Lamé constant	$\eta_{i}$	Corresponding viscosity
E	Young’s modulus	$F_{\mathrm{pre}}$	Preload
ν	Poisson’s ratio	F	Force
E(t)	Time-dependent Young’s modulus	$u_{\max}$	Maximum deformation
τ(t)	Time-dependent stress relaxation	u	Deformation
$\tau_{t}$	Time-dependent viscous component of the stress	Ω	Boundary surface
$t_{b}$	Duration for the load	$t_{h}$	Holding duration
$\dot{\gamma }$	Strain rate tensor		

## Abbreviations

The following abbreviations are used in this manuscript:

SSC	Structural Supercapacitor	DoI	Degrees of Integration
PVA	Polyvinyl Alcohol	MESC	Multifunctional Energy Storage Composites
FEM	Finite-Element Method	FEA	Finite-Element Analysis
GPE	Gel Polymer Electrolyte	RMD	Relative Mean Deviation
